# Equity and Prevention of Cardiovascular Diseases in Latin America and the Caribbean

**DOI:** 10.5334/gh.1123

**Published:** 2022-06-02

**Authors:** Eugenia Ramos, Monica Andreis, Craig Beam, Ronnie Bissessar, Deborah Chen, Marielba Cordido, Javier Valenzuela, Andreas Wielgosz, Nathan Wong

**Affiliations:** 1 InterAmerican Heart Foundation, US; 2 ACT Promoção da Saúde, BR; 3 Trinidad and Tobago heart Foundation, TT; 4 Heart Foundation of Jamaica, JM; 5 Fundación Venezolana del Corazón, VE; 6 University of Ottawa Faculty of Medicine, CA; 7 University of California Irvine, US

**Keywords:** Advocacy, Prevention of Cardiovascular Diseases, Latin America, Caribbean, InterAmerican Heart Foundation

## Abstract

Non-communicable diseases, particularly cardiovascular diseases, are the leading cause of decreased life expectancy and death in Latin America and the Caribbean. Although a lifestyle, which includes no tobacco use, good nutrition, and regular physical activity is touted as key to health, the environmental, racial, social and economic conditions, which underpin lifestyle are often ignored or considered only secondarily. Placing the main responsibility on a patient to change their lifestyle or to simply comply with pharmacological treatment ignores the specific conditions in which the individual lives. Furthermore, there are major disparities in access to both healthy living conditions as well as access to medical care.

There is sufficient evidence to support advocating for policies that support healthy living, particularly healthy food choices. Progress is being made to improve the food environment with enactment of front of package nutritional labels. However, policies were enacted only after intense regional research and advocacy supporting their implementation.

Government officials must rise above the pressures of commercial interests and support health-promoting policies or be exposed as self-interest groups themselves. Strong advocacy is required to persuade officials that all policies should take health into consideration both to improve lives and economies.

Non-communicable diseases (NCD), particularly cardiovascular diseases (CVD), are the leading cause of decreased life expectancy and death in Latin America and the Caribbean (LAC) [1]. Although a lifestyle, which includes no tobacco use, good nutrition, and regular physical activity is touted as key to health, the environmental, racial, social and economic conditions, which underpin lifestyle are often ignored or considered only secondarily. Placing the main responsibility on a patient to change their lifestyle or to simply comply with pharmacological treatment ignores the specific conditions in which the individual lives. Furthermore, there are major disparities in access to both healthy living conditions as well as access to medical care. Access is predicated not only on availability but also on awareness, opportunity, and affordability, with significant disparities in each of these. Some demographic groups are affected more than others, notably Indigenous peoples, those of low socioeconomic status, women, and immigrants who are generally of a racial or religious minority group.

In 2017 the InterAmerican Heart Foundation, with support from the World Heart Federation, initiated a survey of the CVD and NCD policy landscape in the LAC region. The LAC Civil Society Scorecard project, currently being updated, gathered data from 12 countries about main indicators and policies affecting CVD, related NCD, and their main risk factors [2]. The study revealed that few countries measure inequities in morbidity and mortality rates due to NCDs and that a lack of information on care gaps for primary and secondary prevention and for treatment is common. Few countries have information about the proportion of their at-risk population that has periodic medical checkups and/or know their risk factor levels, even after events such as heart attacks. Most countries do not adequately measure the quality of care provided to patients. Only four countries had legislation mandating CVD medicines at fully affordable prices or at no cost, while three had only some provisions in place to address access and cost. These data likely signal widespread inequities and low standards of care that are often not even measured, let alone prioritized for improvement.

There is, however, sufficient evidence to support advocating for policy interventions that ban the marketing of unhealthy foods and beverages to children and inform consumers about critical nutrients such as sugar, salt, saturated and trans fats. However, progress is slow. Expectations are high that governments, whether local, regional, or national, will prioritize and act on policies supporting health. Since ratification of the WHO Framework Convention on Tobacco Control (FCTC) in 2005, many countries in the region have enacted comprehensive tobacco control laws, most recently Bolivia [3]. New laws address health warnings, advertising bans and higher tobacco taxes as well as smoke-free environments in workplaces and public spaces but it is necessary to advance on these policies faster.

Progress is being made to improve the food environment with enactment of front of package nutritional labels (FOPL) in Chile, Ecuador, Peru, Mexico, Uruguay and now Argentina [4]. FOPL effectively convey information to consumers, improving their ability to make healthier choices while also inducing reformulation of products by manufacturers. However, policies were enacted only after intense regional research and advocacy supporting their implementation.

Oftentimes, however, even after being provided with the best scientific evidence and strong recommendations, governments succumb to pressures from commercial interest groups blocking or reversing important public health decisions. In Jamaica, an earlier vote supporting the ‘high in’ octagon FOPL model was reversed, likely due to industry interference. This decision was upheld by the Cabinet, leading Jamaica to vote against it [5]. In Brazil, the inter-ministerial commission in charge of coordinating FCTC implementation was excluded from the 9^th^ meeting of the Conference of the Parties to the FCTC this past year [6].

Government officials must rise above the pressures of commercial interests and support health-promoting policies or be exposed as self-interest groups themselves.

Finally, another area of yet unmitigated inequities is the early detection and treatment of comorbidities associated with CVD. Unequal access, availability and cost of newer treatments are enduring barriers that have challenged countries in the region for decades.

Inequities must be addressed to meet the SDG 2030 goals [7]. With governments focused on economic priorities and limiting expenditures, budgets for health are at risk of being significantly curtailed. Furthermore, the return on investment in health is longer term and often too long to secure re-election, which is a priority for elected government officials. Therefore, strong advocacy is required to persuade officials that all policies should take health into consideration both to improve lives and economies.
